# Determinants of acceptance of cervical cancer screening in Dar es Salaam, Tanzania

**DOI:** 10.1186/1471-2458-12-1093

**Published:** 2012-12-19

**Authors:** Crispin Kahesa, Susanne Kjaer, Julius Mwaiselage, Twalib Ngoma, Britt Tersbol, Myassa Dartell, Vibeke Rasch

**Affiliations:** 1 Department of International Health, Immunology and Microbiology, University of Copenhagen, Copenhagen K, Denmark; 2 Ocean Road Cancer Institute, Dar es Salaam, Tanzania; 3 Department of Virus, Lifestyle and Genes, Institute of Cancer and Epidemiology, Danish Cancer Society Gynaecologic Clinic Rigshospitalet, University of Copenhagen, Copenhagen K, Denmark; 4 Department of Gynaecology and Obstetrics, Odense University Hospital, Denmark

**Keywords:** Cervical cancer, Screening acceptance, Demographic characteristics, Knowledge, Tanzania

## Abstract

**Objective:**

To describe how demographic characteristics and knowledge of cervical cancer influence screening acceptance among women living in Dar es Salaam, Tanzania.

**Methods:**

Multistage cluster sampling was carried out in 45 randomly selected streets in Dar es Salaam. Women between the ages of 25–59 who lived in the sampled streets were invited to a cervical cancer screening; 804 women accepted and 313 rejected the invitation. Information on demographic characteristics and knowledge of cervical cancer were obtained through structured questionnaire interviews.

**Results:**

Women aged 35–44 and women aged 45–59 had increased ORs of 3.52 and 7.09, respectively, for accepting screening. Increased accepting rates were also found among single women (OR 2.43) and among women who had attended primary or secondary school (ORs of 1.81 and 1.94). Women who had 0–2 children were also more prone to accept screening in comparison with women who had five or more children (OR 3.21). Finally, knowledge of cervical cancer and awareness of the existing screening program were also associated with increased acceptance rates (ORs of 5.90 and 4.20).

**Conclusion:**

There are identifiable subgroups where cervical cancer screening can be increased in Dar es Salaam. Special attention should be paid to women of low education and women of high parity. In addition, knowledge and awareness raising campaigns that goes hand in hand with culturally acceptable screening services will likely lead to an increased uptake of cervical cancer screening.

## Background

With an estimated 500,000 new cases and the cause of 273,000 deaths each year, cervical cancer is one of the most prevalent and deadly female cancers worldwide. The vast majority of cervical cancer cases (99,7%) are linked to genital infection with human papillomavirus (HPV) [1], a common virus that is sexually transmitted [2]. Sub-Saharan Africa is by far the most affected region, accounting for 80% of the new cases and 85% of the deaths from cervical cancer worldwide [3]. In developed countries with well-established screening programs, the incidences of cervical cancer have been reduced by 70-90% [4,5]. In contrast, in developing countries where access to screening services for cervical cancer is often limited or nonexistent the incidence of women affected by the disease continues to exist at high levels [6-8].

In the developed world, the introduction of pap smear as a screening test modality has led to a reduction of the burden of cervical cancer. However, there are several factors that, in addition to the availability of a screening test, contribute to reducing the burden of cervical cancer. These include prevalence rates of HPV, effective screening strategies, availability of facilities for diagnostic follow up and prompt treatment of detected lesions. The participation rate in cervical cancer screening is of utmost importance for the effectiveness of a cervical cancer screening program [9,10]. The adoption of pap smear as a screening approach in the developing world has been found to be impractical due to the lack of trained cytotechnologists, cytology laboratories and inefficient health systems [5,11]. In an effort to curb the burden of cervical cancer in developing countries, the World Health Organization (WHO) and the International Agency for Research on Cancer (IARC) have established cervical cancer screening programs by adopting an alternative screening method based on visual inspection with acetic acid (VIA) [12,13]. The VIA test has proven to have similar sensitivity to that of cytology but lower specificity and positive predictive value when evaluated in clinical research settings [14]. Experiences from Tanzania indicate that when VIA test is introduced for wide-spread routine use it may be at the cost of poorer test performance [15].

When focusing on Tanzania, cervical cancer is, with an estimated incidence rate of 68.6/100,000, the leading cause of cancer and cancer-related deaths [16]. This incidence rate is also high when compared to other sub-Saharan African countries. At Tanzania’s only cancer center, the Ocean Road Cancer Institute in Dar es Salaam, 80% of cervical cancer patients have already progressed to a late, incurable stage by the time women present themselves for medical care. Acknowledging the increasing burden of the disease, a cervical cancer screening program based on VIA test was established in Dar es Salaam with support from WHO, IARC and the International Network for Cancer Treatment and Research in 2002. An evaluation of the program documented that the VIA test was effective as a screening test for cervical cancer prevention and it was decided to offer VIA testing for routine use [17]. The routine program is targeting approximately 500,000 women living in Dar es Salaam. Five years after project implementation, screening attendance was evaluated and it was found that only 4% of the target population had been screened. To address the poor screening coverage, the cervical cancer screening program in Tanzania has undergone several iterations in recent years. Despite these efforts, the available screening resources are still not utilized by the vast majority of women living in Dar es Salaam [18].

To inform the strategy for future scale-up of cervical cancer screening and better reach women with cervical cancer screening services, this paper focuses on a group of women who were invited to attend the cervical cancer screening program in Dar es Salaam and describes how demographic characteristics and knowledge of cervical cancer are associated with screening acceptance.

## Methods

### Study setting

**Figure 1:**
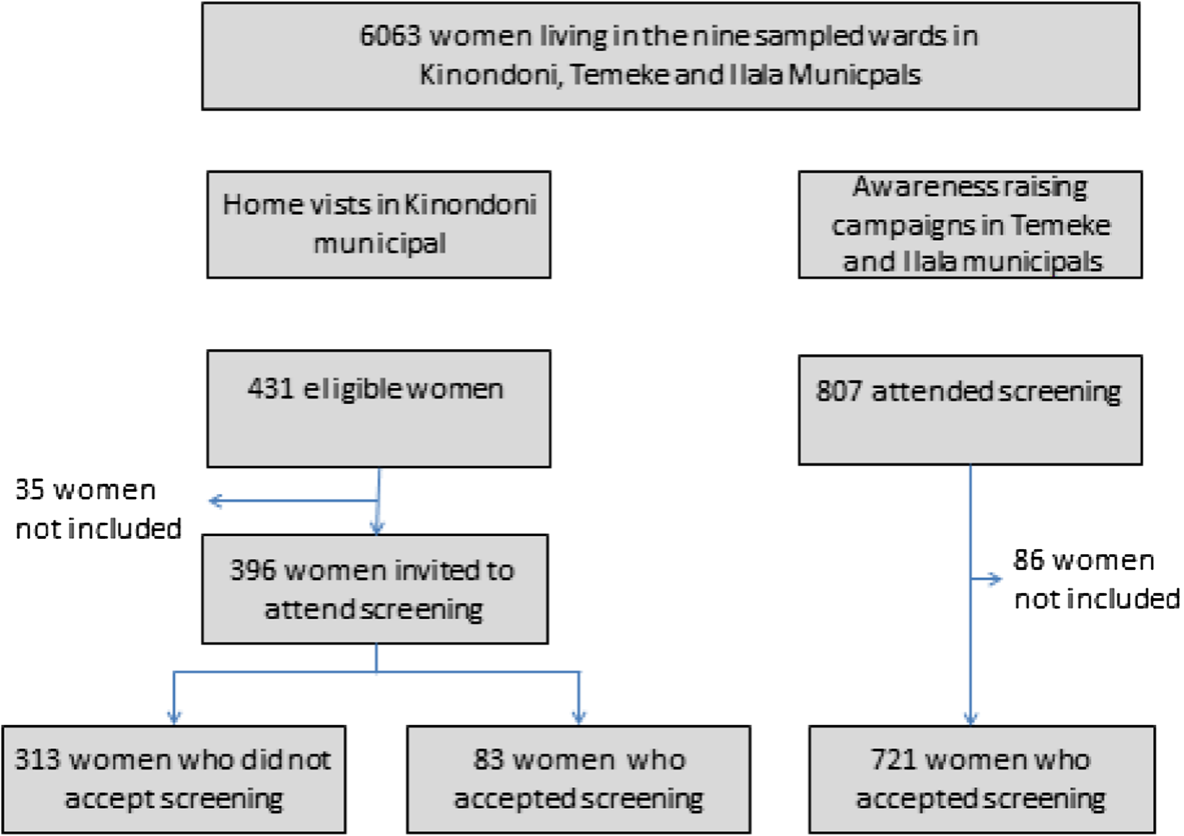
Schematic view of the study population.

The study was carried out among women aged between 25–59 years living in Dar es Salaam, Tanzania. Based on the 2002 Population and Housing Census, Dar es Salaam had 2,487,288 inhabitants, of whom 700,000 were women in a reproductive age group. Ninety-five percent of the residents are engaged in the non-formal sector and the rest are formally employed. Women are mainly involved in the domestic sphere and the literacy level is more than 50%. The city is divided into three municipal councils: Kinondoni, Ilala and Temeke. The population is served by one national hospital, three municipal hospitals and a number of private hospitals and dispensaries. Additionally, Dar es Salaam has the only centre for specialized cancer care in Tanzania, Ocean Road Cancer Institute. The study population comprised of a group of women from the general population who were aged 25–59 years and who had never attended cervical cancer screening (Figure 1).

### Study population

To identify a representative group of women, multistage cluster sampling was performed where three wards from each of the three municipals in Dar es Salaam were randomly selected. In all 6063 women in the age group 25–59 years were living in the nine sampled wards. Five streets were subsequently selected randomly from each of the wards. Two strategies were employed to recruit the study participants. The first approach was home visits performed by the principal investigator and research assistants with help from the community leaders from the respective streets in Kinondoni municipal. In all 431 women were considered eligible for the study, 35 of these women were not included in the study since they were either not at home when attempted visited or stated they had previously attended screening. The remaining 396 women were interviewed and afterwards provided with health education on cervical cancer and invited to attend screening at Ocean Road Cancer Institute. Only 83 women accepted the invitation. The second approach relied on combination of outreach services and awareness raising campaigns. This approach was used in the two remaining municipals, Temeke and Ilala. Through campaigns that were conducted by means of megaphones in the sampled streets, women were provided with health information and invited to attend outreach screening for cervical cancer at the municipal hospitals. In all, 807 women showed up for screening, 86 of these women were excluded from the study sample because they were either living outside the selected streets (n=41) or had previously attended screening (n=45).

### Data collection and analyses

Structured questionnaire interviews focusing on demographic characteristics and knowledge and awareness of cervical cancer were performed among the 1117 women who were either visited at home and invited to attend screening or had attended screening after awareness raising campaigns. To assess how demographic characteristics were associated with screening acceptance, we compared age, marital status, educational level and number of children between the 804 women who accepted screening after either home visit or awareness raising campaigns and the 313 women who did not accept the screening invitation. The association between screening acceptance and knowledge and awareness of cervical cancer was determined through a comparison of the 83 women from the home visit group who accepted screening and the 313 women from the home visit group who did not accept the screening invitation.

Data were entered in EPIINFO version 6.04 and then exported to SPSS version 13 for analysis. Crude odds ratios (ORs) with 95% confidence interval (CI) were calculated where the women’s screening acceptance comprised the dependent variable and demographic characteristic and factors related to the women’s knowledge of cervical cancer the independent variables. Adjusted analysis was performed by means of multiple logistic regression, where the influence of age, marital situation, parity and educational level were controlled for.

Permission to carry out the study was obtained from the Tanzania National Institute of Medical Research and the Danish National Committee on Biomedical Research Ethics. Verbal informed consent to participate in the study was obtained from participants. Participants who were found to have medical problems were assisted to visit the referral hospital for further management and treatment.

## Results

The demographic characteristics of the general population together with the demographic characteristics of the 83 women who accepted screening after home visits and the 721 women who accepted screening after awareness raising campaigns are summarized in Table 1. Women who attended screening after home visits or awareness raising campaigns were more often aged 45–59 (21% and 23%, respectively), were more often married (80% and 78%, respectively) and had more often attended secondary school (58% and 33%, respectively) in comparison with the general population where the corresponding figures were 12%, 63% and 16%. In addition, the women who accepted screening after home visits had more often given birth 5 times or more (39%) than women from the general population (21%). In contrast, women who accepted screening after awareness raising campaigns had more often given birth twice or less (44%) when compared to the general population (34%).

**Table 1:** Demographic characteristics of the general population and women who accepted the cervical cancer screening invitation via the home visit approach and awareness raising campaign

	**General population N=6063 %**		**Home visits N=83 %**		**Awareness raising N=721 %**	
**Age(years)**						
25-34	3092	51.0	38	45.8	281	38.9
35-44	2231	36.8	28	33.7	273	37.9
45-59	740	12.2	17	20.5	167	23.1
**Marital status**						
Married	3844	63.4	66	79.5	562	77.9
Single	2219	36.6	17	20.5	159	22.1
**Education**						
No schooling	1158	19.1	6	7.2	43	6.0
Primary school	3923	64.7	29	34.9	442	61.3
Secondary school	982	16.2	48	57.8	236	32.7
**No of children**						
0-2	2061	34.0	8	9.6	316	43.8
3-4	2728	45.0	43	51.8	259	35.9
5+	1274	21.0	32	38.6	146	20.3

The associations between screening acceptance and demographic characteristics are summarized in Table 2. Women aged 35–44 and women aged 45–59 had increased ORs of 3.52 and 7.09, respectively, for accepting the invitation in comparison with women aged 25–34. Similarly, married women had an increased OR 2.43 for accepting the screening invitation. Increased ORs for accepting screening were found among women who had attended primary or secondary school in comparison with women who had never attended screening (OR 1.81 and OR 1.94, respectively). Finally, women who had 0–2 children were more prone to accept the invitation in comparison with women who had five or more children (OR 3.21).

**Table 2:** Comparison of demographic characteristics of women who accepted and who did not accept a cervical cancer screening invitation

	**Accepted screening invitation N=804 %**		**Did not accept screening invitation N=313 %**		**Accepted vs. non accepted Crude OR (95%CI)**	Accepted vs. non accepted Adjusted* OR (95%CI)
**Age(years)**						
25-34	319	39.7	153	48.9	1	1
35-44	301	37.4	104	33.2	1.39 (1.02-1.88)	3.52 (2.37-5.14)
45-59	184	22.9	56	17.9	1.58 (1.09-2.29)	7.09 (4.19-12.3)
**Marital status**						
Married	628	78.1	214	68.4	1.18 (0.82-1.84)	2.43 (1.18-2.20)
Single	176	21.9	51	16.3	1	1
**Education**						
No schooling	49	6.1	35	11.2	1	1
Primary school	471	58.6	208	66.4	1.62 (0.99-2.63)	1.81 (1.01 - 3.42)
Sec. school	284	35.3	70	22.4	2.90 (1.69-4.96)	1.94 (1.13 - 4.01)
**No of children**						
0-2	324	40.3	54	17.2	3.74 (2.53-5-53)	3.21 (1.71-7.03)
3-4	302	37.6	129	41.2	1.46 (1.05-2.02)	1.08 (0.98-2.71)
5+	178	22.1	111	35.4	1	1.00

*Adjusted for the effect of age, education, marital status and number of children.

The 396 women, who in relation to the home visit stated they had never attended screening, were questioned about their knowledge of cervical cancer and perceived barriers for attending health check-ups (Table 3). More than half (53%) of the women had never heard of cervical cancer. When questioned about perceived barriers for attending health check-ups, 57% stated that difficult access to health service would be a hindering factor. In addition, 31% of the women stated they were reluctant to go for any test in absence of disease. Lack of medical advice and fear of being diagnosed as having cancer were additionally mentioned as a barrier by 12% and 13% of the women.

**Table 3:** Knowledge of cervical cancer and perceived barriers for attending health check ups among women who had never attended cervical cancer screening

	**N=396**	**Percentage (%)**
**Have ever heard of cervical cancer**		
Yes	187	52.8
No	170	47.2
Missing	39	
Perceived barriers for attending health check-up*		
Difficulty in accessing screening services	226	57.1
Lack of health education	34	8.5
Reluctance to go for any test in absence of disease	123	31.1
Lack of medical advice	46	11.6
Fear of knowing they have cancer	50	12.6
Prohibitive cost of the test	8	2.0
Fear of pain of test	19	4.8

*Add more than 100% since the women were able to respond to more than one question.

Table 4 summarizes the association between screening acceptance and the women’s knowledge and awareness of cervical cancer. Awareness of cervical cancer and screening service were positively associated with screening acceptance (ORs 5.90 and 4.20, respectively). Similarly, the women’s knowledge of cervical cancer risk factors was also found to be a determining factor for screening attendance (ORs of 3.38). Finally, women who believed that cervical cancer could be prevented and women who believed screening could improve survival were also more likely to accept screening with increased ORs of 10.1 and 10.4, respectively.

**Table 4:** Knowledge of cervical cancer screening service among women who accepted and women who did not accept a cervical cancer screening invitation

	**Accepted screening invitation N=83 (%)**		**Did not accept screening invitation N=313 (%)**		**Accepted vs. non accepted Crude OR (95%CI)**
**Heard about cervical cancer**					
Yes	68	81.9	119	43.4	5.90 (3.1-11.38)
No/Don’t know	15	18.1	155	56.6	1
Missing	0		39		
**Heard about screening**					
Yes	62	74.7	116	41.3	4.20 (2.35-7.56)
No/Don’t know	21	25.3	165	58.7	1
Missing	0		32		
**Knowledge of cervical cancer risk factors**					
Yes	38	45.8	62	20.0	3.38 (1.96-5.83)
No/Don’t know	45	54.2	248	80.0	1
Missing	0		3		
**Heard of HPV**					
Yes	4	4.8	10	3.2	1.52 (0.39-5.45)
No/Don’t know	79	95.2	300	96.8	1
Missing	0		3		
**Can cervical cancer be prevented**					
Yes	24	28.9	12	3.9	10.1 (4.55-22.9)
No/Don’t know	59	71.1	299	96.1	1
Missing	0		2		
**Can screening improve survival?**					
Yes	56	67.5	52	16.6	10.4 (5.82-18.7)
No/Don’t know	27	32.5	261	83.4	1
Missing	0		0		

## Discussion

As cervical cancer screening is being increasingly implemented in developing countries, there is a need to consider potential determinants of acceptance of cervical cancer screening in such settings. The present study is based on data from Tanzania and reveals that screening acceptance is associated with being older, being married, having attended school, and having less than 5 children. Furthermore, knowledge and awareness of cervical cancer and screening benefits seem to have a positive impact on screening acceptance.

This study was designed to include a representative group of women from a population that had not previously attended cervical cancer screening and then assess how they responded to a screening invitation via different approaches. Thereby the study has shed light on the effect of different strategies for inviting women to cervical cancer screening. Home visits lacked efficiency in improving screening attendance due to its low acceptance rate. In contrast, the approach of decentralizing cervical cancer screening to district level and combining the decentralization with awareness raising campaigns was quite effective in making women accepting screening. Hence, cervical cancer screening attendance may be enhanced if a move is made from the current centrally organized screening system to a more decentralized system where the service is offered at a few selected antenatal clinics or by mobile out-reach clinics.

The findings from the present study suggests that older women are more responsive to accept screening than younger women. A finding which is well in line with the recommendation that cervical cancer screening programs should aim at targeting older women since the benefit from screening this group of women is particularly high. It has for instance been demonstrated that participation in the UK cervical screening program by women aged 35–64 reduces the risk of cervical cancer over the next five years by 60-80% and the risk of advanced cervical cancer by about 90% [19]. In addition, the relative protection against cervical cancer is higher in older women than in women aged 20–34 years [19]. Women of high parity comprised an identifiable subgroup that was less likely to attend screening in the setting studied. A finding which should raise concern since the enrolment of high parity women in cervical cancer screening programs is particularly important given that high parity is known to be a co-factor of HPV carcinogenesis via mechanical, hormonal and immunological mechanisms [20-22]. A possible explanation for the low screening acceptance among high-parity women may be that practical barriers such as foregoing domestic activities are more prevalent among this group of women. To reach the women most at risk of cervical cancer it is of paramount importance that reproductive health programs strive to encourage screening among high parity women. In that relation it should be stressed that home visits apparently were more effective than awareness raising campaigns in making women of high parity attend screening. Educational level did also seem to matter for women’s screening acceptance. Women who had attended at least primary school were more likely to attend screening in comparison with women who had never attended school. These findings are in line with other studies that have documented that women with low education are less knowledgeable about the need for cervical screenings and have limited resources to cater for their screening attendance due to its inherent cost [23]. In addressing women’s needs, it should be acknowledged that many women, in addition to economic constraints, counter problems such as long waiting time, cultural deterrents to care and poor quality of health services when attempting to access screening services. Attempts should be made to eliminate these barriers when scaling up cervical cancer screening. In addition, since wide spread routine use of VIA testing may be associated with poorer test performance [15,24] more sensitive, objective and reproducible screening tests should be considered when scaling up cervical cancer screening. HPV testing in combination with VIA testing, where the VIA test function as a triage/treatment activity following HPV testing may be more appropriate for cervical cancer screening in developing countries [24]. One of the drawbacks with the inclusion of HPV as a primary screening method is the associated costs. However, with an increasing availability of simple, affordable, and accurate HPV tests (careHPV test, Qiagen Gaithersburg, Inc. MD, USA) that provides results within 3 hours [25] it has been suggested that cervical cancer screening in low-resource settings increasingly should be supplemented by HPV testing [24].

The knowledge and awareness of cervical cancer was in general low and screening acceptance was associated with having knowledge of cervical cancer, its risk factors and, its prevention. Poor knowledge of cervical cancer has also been found in the more general population of Tanzania [26]. A number of studies from other sub-Saharan African countries have similarly found that women who lack awareness of cervical cancer are less likely to participate in screening services and are thus at increased risk of developing cancer [27-29]. In our study almost half of the women (47%) had never heard the term cervical cancer. This lack of biomedical knowledge may partly be explained by the fact that cervical cancer, despite being the most common female cancer in sub-Saharan Africa, is a rare condition that has not been prioritized by the national health system, advocacy programs have therefore not focused on cervical cancer [30]. More diverse strategies should be employed to convey educational health messages which take into account the women’s socio economic and cultural background. In that relation it should be born in mind that experiences from both developed and developing countries have shown that conveying message via word of mouth and via audio visual channels are effective in making women more aware of cervical cancer and screening possibilities [31]. In addition, health education through trained lay persons in community centers should also be considered as this has been reported to be an effective method in other studies [32,33].

In conclusion, we have documented that there are identifiable subgroups among which cervical cancer screening can be increased in Dar es Salaam. Special attention should be paid to poorly educated women and women of high parity. Lack of knowledge of cervical cancer also contributed in preventing women from attending cervical cancer screenings. Women’s perceptions and notions about cervical cancer need to be further assessed to develop communication strategies that take a broader cultural framework into account. Providing education and information orally as well as improving access to more culturally acceptable screening services will likely lead to increased uptake of screening services in Tanzania.

## Competing interests

KKS received lecture fees, advisory board fees, and research grants through her institution from Merck and Sanofi Pasteur MSD.

## Authors’ contributions

KC participated in the conception, design, and implementation of the study, statistical analysis, interpretation and drafting of manuscript. KKS and RV participated in developing the study design, the implementation and the interpretation of the findings. DM, MJ, NT and TB participated in developing the study design and assisted with implementation of the study. All authors have read and approved the final manuscript.

## Pre-publication history

The pre-publication history for this paper can be accessed here:

http://www.biomedcentral.com/1471-2458/12/1093/prepub
